# Correction to *Lancet Glob Health* 2016; 4: e633–41

**DOI:** 10.1016/S2214-109X(16)30340-0

**Published:** 2016-12-10

*Do NTT, Ta NTD, Tran NTH, et al. Point-of-care C-reactive protein testing to reduce inappropriate use of antibiotics for non-severe acute respiratory infections in Vietnamese primary health care: a randomised controlled trial.* Lancet Glob Health *2016; **4:** e633–41*—In this Article (September, 2016), the copyright licence should have been CC BY. This correction was made on Dec 9, 2016.

